# Psychological resilience and depressive symptoms among family caregivers of disabled people: the mediating role of caregiver burden and the moderating role of family functioning

**DOI:** 10.3389/fpsyg.2026.1852506

**Published:** 2026-08-14

**Authors:** Zhuoya Yang, Kexin Wei, Chong Wang, Yitong Liu, Yuexia Gao, Miaomiao Zhao, Fang Lu, Yaqin Zhong

**Affiliations:** 1 School of Public Health, Nantong University, Nantong City, China; 2 Institute for Health Development, Nantong University, Nantong, China; 3 Wuxi No.8 People’s Hospital, Wuxi, China

**Keywords:** caregivers, psychological resilience, depressive symptoms, disabled people, moderated model

## Abstract

**Background:**

Family caregivers of disabled older adults frequently face significant challenges that are associated with their mental health. Psychological resilience and family functioning may be associated with depressive symptoms among this population. Nevertheless, the mechanisms behind this relationship have received limited attention. In this study, we examined whether caregiver burden serves as a mediating factor and family functioning as a moderating factor in the link between psychological resilience and depressive symptoms among family caregivers of individuals with disabilities.

**Methods:**

A community-based cross-sectional survey was conducted in Nantong and Taizhou, eastern China, from December 2023 to May 2024. Data were collected using the 10-item Connor-Davidson Resilience Scale, the 12-item Zarit Burden Interview, the 10-item short form of the Center for Epidemiological Studies Depression Scale, and the Family APGAR Scale. A moderated mediation model was tested using bootstrap and simple slope procedures.

**Results:**

A total of 353 caregivers completed the survey. The prevalence of depressive symptoms was 31.59%. Higher psychological resilience was significantly associated with fewer depressive symptoms. This relationship was partially mediated by caregiver burden. In addition, family functioning moderated the mediated relationship, indicating that the indirect effect of psychological resilience on depressive symptoms via caregiver burden varied across levels of family functioning.

**Conclusion:**

These findings suggest that psychological resilience, caregiver burden, and family functioning are jointly associated with depressive symptoms in family caregivers of disabled older adults. Interventions targeting psychological resilience and family functioning may be associated with reduced caregiver burden and depressive symptoms in this population.

## 1 Introduction

Population aging is a dominant demographic trend linked to increased disease risks. In China, over 42 million older adults are disabled, and this number is projected to reach 52.24 million by 2050 (Cao, 2019). Disability is associated with poor physical health, chronic conditions, and reduced quality of life, posing challenges to families and healthcare systems (Lin et al., 2025). Such functional decline also heightens psychological vulnerability by limiting social resources and shaping emotional responses to support (Zhang et al., 2025). Given the growing disabled population, mitigating depressive symptoms among older adults and their caregivers has become a public health priority.

The high prevalence of depressive symptoms among family caregivers of disabled older adults highlights considerable public health implications of this research topic. A study of 561 caregivers in Zunyi reported a depression prevalence of 44.21% (Jiao et al., 2023), indicating that clinically significant depressive symptoms are widespread among this caregiver population and far exceed those observed in the general older population. This elevated psychological distress among caregivers echoes the notion that disability functions as a restrictive context, wherein the mental health burden extends beyond the affected individuals to permeate the entire family system (Zhang et al., 2025).

The rising disabled population has intensified caregiver burden. Disability imposes financial strain and hardship on families. In China’s cultural context, filial piety, a central norm obligating children to care for aging parents, shapes caregiving arrangements and psychological experiences (Zhao, 2023). Most disabled older adults prefer family care over institutional settings, making family caregiving the primary support model. The dual filial piety model distinguishes reciprocal (affection-based) from authoritarian (obedience-based) piety (Yeh and Bedford, 2003). Reciprocal piety tends to protect against depression by fostering meaningful caregiving relationships, whereas authoritarian piety may lead to adverse outcomes when caregivers feel trapped between cultural expectations and unsupportive family environments (Zhang et al., 2024). Culturally, family-based eldercare expectations intensify perceived burden (Qiu et al., 2020), and intergenerational co-residence may amplify relational tensions under high care demands (Zhao, 2023). Thus, filial piety may heighten the burden when family functioning is poor, yet strengthen resilience when caregiving is reframed as a meaningful cultural expression. These cultural dynamics underscore the need to examine how psychological resilience interacts with family functioning in the Chinese caregiving context. This long-term, unpaid care is a chronic stressor that harms caregivers’ mental health (Qu et al., 2022). Therefore, understanding the pathways underlying care-related depression is essential for developing effective interventions.

### 1.1 Psychological resilience and depressive symptoms

Psychological resilience is recognized as a significant contributor to psychological wellbeing (Parviniannasab et al., 2024). It is a complex and multidimensional construct (Hagedoorn et al., 2008), referring to the ability of some individuals to withstand adversity and overcome difficulties. According to the resilience framework, resilience operates as a dynamic process that fosters personal growth in challenging circumstances through various mediating mechanisms (Kumpfer, 2002). In the context of chronic stress, such as that experienced by family caregivers of disabled older adults, resilience confers protective effects against depression through several pathways. It enhances adaptive coping by promoting problem-focused strategies and help-seeking while reducing avoidant coping that exacerbates distress. It also facilitates positive cognitive appraisal, enabling caregivers to reframe daily burdens as meaningful challenges. Resilient individuals show stronger emotional regulation, allowing them to sustain emotional homeostasis amid caregiving-related stress. Finally, resilience is associated with more efficient stress recovery, mitigating cumulative wear-and-tear that contributes to depressive symptomatology (Tugade and Fredrickson, 2004). Evidence suggests that resilience plays a protective role by improving individuals’ capacity to cope with and adapt to stressful situations (Bogner et al., 2012). A longitudinal study by Wu et al. (2017) found a significant negative association between baseline resilience and depressive symptoms assessed one year later. Similarly, Kara Ar and Canli (2020) reported an inverse relationship between resilience and depression. However, most existing research has focused on general or student populations, leaving the specific context of family caregivers of disabled older adults largely unexplored. The protective role of resilience in this highly stressed group remains poorly understood and warrants further investigation.

### 1.2 The potential mediators between psychological resilience and depressive symptoms

Caregiver burden is defined as the challenges experienced by caregivers, including a sense of overwhelming responsibility, persistent worry, and restrictions on caregivers’ social interactions (Charbaji et al., 2026). On the one hand, faced with substantial caregiving burdens, psychological resilience is critical for mitigating adverse effects. Research indicates that reduced resilience correlates with increased caregiver burden (Ji et al., 2025). The higher the resilience, the lighter the burden (Peng et al., 2019). On the other hand, studies have established a significant link between caregiver burden and depressive symptoms. One study showed that caregiver burden predicts depressive symptoms (Wrede et al., 2025). Another found a strong positive correlation between subjective burden and depression among family caregivers of older adults (Del-Pino-Casado et al., 2019). Caregivers with more severe burden are more likely to experience depressive symptoms, with symptoms worsening over time. This association may stem from financial strain, extended caregiving hours, and restricted social interactions. Both psychological resilience (Awano et al., 2020) and caregiver burden (Ren et al., 2017) can influence depressive symptoms.

To clarify the theoretical underpinnings of these relationships, the Stress Process Model was adopted. This model conceptualizes the caregiving experience as a hierarchical framework consisting of four interrelated domains, background and contextual factors, primary stressors, secondary role strains, and mental health outcomes (Pearlin et al., 1990). Primary stressors denote the objective demands directly implicated in care provision, whereas secondary strains encompass the broader ripple effects of caregiving across other life domains. Critically, the model posits that the influence of stressors on psychological outcomes is contingent upon the coping resources available to the caregiver, which may mediate or moderate the stress outcome pathway. Although extensive research has examined competing mediating pathways encompassing perceived stress, coping strategies, social support, caregiving self-efficacy, and emotional exhaustion (Pinquart and Sörensen, 2003), caregiver burden was chosen due to its primacy within the theoretical framework of the Stress Process Model. According to Pearlin et al. (1990), primary stressors are the most direct consequence of caregiving demands, whereas secondary strains and coping resources operate at later stages. Empirical evidence suggests that caregiver burden predicts mental health outcomes more directly than coping strategies or social support, which tend to exert effects indirectly through burden (Kim and Carver, 2012; Li et al., 2014). Furthermore, resilience, as an inherent personal resource, is theorized to modulate individuals’ subjective appraisal of primary caregiving stressors (namely, caregiver burden) before exerting impacts on subsequent psychological outcomes such as emotional exhaustion and perceived stress (Tugade and Fredrickson, 2004). Accordingly, caregiver burden was selected as the mediator. Within this framework, burden represents perceived caregiving demands (primary stressor), depressive symptoms serve as the mental health outcome, and resilience shapes how caregiving demands are appraised and experienced. Thus, resilience is hypothesized to reduce depressive symptoms indirectly by alleviating perceived burden. Nevertheless, limited empirical research has quantitatively examined this mediating pathway among family caregivers of disabled older adults. The present study therefore examined the hypothesis that higher resilience would be associated with lower burden, which in turn would be associated with fewer depressive symptoms.

### 1.3 The potential moderators between psychological resilience and depressive symptoms

Family functioning refers to how family members interact and collaborate (Li et al., 2023). According to family systems theory, well-functioning families have close relationships, healthy emotional expression, mutual understanding, strong cohesion, and better problem-solving capacity (Desjardins et al., 2022; Liu et al., 2022). These features help reduce negative emotions. Prior research shows that family functioning affects depressive mood (Dang et al., 2026; Zhang and Gao, 2023). Good family functioning provides emotional support and stability, which reduces depressive symptoms (Guerrero-Mu Oz et al., 2021). Poor family functioning increases psychological strain and depression risk (Liu et al., 2026). Moreover, family functioning influences how individuals perceive burden (Andrade et al., 2021; Tang et al., 2022). Positive family functioning enhances family resilience and coping, helping to manage the burden and reduce stress outcomes. To support the moderating role of family functioning, we use two frameworks: Hill’s ABC-X model and resource substitution theory. Hill’s ABC-X model (Hill, 1958) identifies four factors in stress outcomes: stressors (A), resources (B), perceptions (C), and crisis (X). In caregiving, long-term demands are the stressor (A), family functioning is a family-level resource (B), and depressive symptoms are the potential crisis (X) (Brik and Wang, 2025). Resource substitution theory (Ross et al., 2001) suggests that when external resources are scarce, people rely more on internal resources; when external resources are abundant, they may replace internal ones. Here, family functioning is external, and psychological resilience is internal. When family functioning is low, caregivers lack external support, so internal resilience becomes critical. When family functioning is high, external support may reduce the protective effect of resilience alone. From a social network perspective, family caregiving is a system of strong ties that provides critical support under chronic stress (Wang et al., 2024). Population aggregation and residential density have been shown to foster strong ties, which in turn mediate the relationship between environmental factors and depression (Wang et al., 2024). Well-functioning families form an effective strong-tie network that shares care demands and strengthens coping. Poor family functioning means this network fails, leaving caregivers to rely on their own resources. This strong-tie perspective helps explain how family functioning moderates caregiving stress pathways.

Based on these theoretical frameworks, we propose that family functioning serves as a dual moderator in the caregiving stress process. First, it may moderate the resilience-burden relationship. When family functioning is high, external support reduces the need for resilience to buffer burden. When family functioning is low, caregivers rely more heavily on resilience to manage burden (Cui et al., 2024). Well-functioning families provide support that complements coping capacity (Li et al., 2022). Conversely, conflict-ridden families lack supportive feedback, making it difficult for even resilient individuals to reduce burden (Cui et al., 2024). Second, family functioning may moderate the burden-depression relationship. Well-functioning families prevent burden from translating into depressive symptoms. Poorly functioning families lack this buffering capacity, allowing burden to escalate into depression (Li et al., 2022). Open communication alleviates distress, coordinated care reduces role overload, and family cohesion fosters meaning and belonging that buffer psychological toll (Pires et al., 2022). In contrast, conflict-ridden families amplify the burden-depression association by adding family tension to care demands (Margolin and Gordis, 2000).

### 1.4 Research hypotheses

Currently, limited research has comprehensively examined the relationships among family functioning, psychological resilience, caregiver burden, and depressive symptoms in family caregivers of disabled older adults within the Chinese context. Understanding these mediating processes is critical for developing interventions that may prevent caregivers from developing or worsening depressive symptoms due to persistent burden in daily caregiving situations.

Although psychological resilience protects against depression in various populations, its specific pathways among family caregivers of disabled older adults remain unclear. Existing studies have focused on general or patient populations rather than caregivers exposed to chronic caregiving stress. Caregiver burden and family functioning have each been linked to depressive symptoms, but most prior research examined bivariate associations, leaving integrated mechanisms less understood. Whether caregiver burden mediates the resilience-depression relationship and whether family functioning moderates this process remain unclear. In addition, recent domestic studies have examined these relationships in Chinese caregiving populations (Niu et al., 2025). Zhu et al. (2026) found that family functioning was associated with depressive symptoms through the serial mediation of perceived stress and resilience among Chinese nursing home residents. However, these studies have largely examined simple mediation or isolated moderation effects, and none have integrated caregiver burden as the mediator and family functioning as the moderator within a single framework for this population.

**Figure 1:**
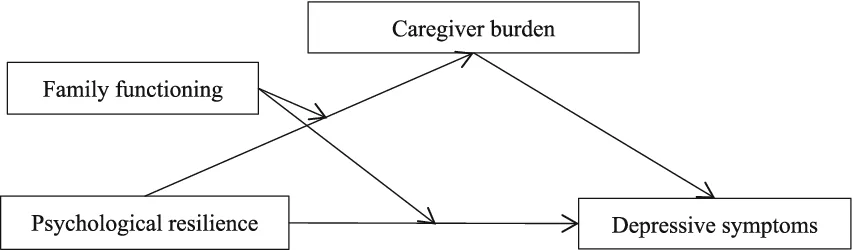
Hypothesized moderated mediation model.

This study addresses these gaps by proposing and testing a moderated mediation model (Figure 1) that delineates the pathway from psychological resilience to depressive symptoms. Specifically, we investigate (a) the mediating role of caregiver burden, and (b) the moderating role of family functioning among Chinese caregivers of disabled older adults. Theoretically, we integrate the Stress Process Model, Hill’s ABC-X model, and strong-tie perspectives to provide a comprehensive framework, extending prior work that examined these factors in isolation. Practically, our findings may inform culturally tailored interventions targeting both individual psychological resources and family-level support, offering actionable insights for healthcare providers and community workers. Additionally, we include exploratory subgroup analyses stratified by gender, age, marital status, and educational attainment. These analyses were not guided by pre-specified hypotheses and are intended solely for descriptive purposes to generate future research directions. Results should be interpreted with caution. The following hypotheses were formulated:

*H1*: Psychological resilience serves as a significant negative predictor of depressive symptoms.

*H2*: The relationship between psychological resilience and depressive symptoms is mediated by caregiver burden.

*H3*: Family functioning moderates the associations among psychological resilience, caregiver burden, and depressive symptoms.

## 2 Methods

### 2.1 Sampling and data collection

This study employed a multistage sampling approach in Nantong and Taizhou, central Jiangsu, between December 2023 and May 2024. Both cities have intermediate levels of economic development and elderly welfare resources relative to the province. Southern Jiangsu (e.g., Suzhou, Wuxi, Changzhou) has well-established formal long-term care systems, whereas northern Jiangsu lags in per capita GDP and healthcare infrastructure. Nantong is a recognized longevity region with life expectancy considerably higher than the national average, implying longer caregiving durations, more recipients aged ≥80, and greater long-term care demands. The sampling procedure was as follows: (1) Four districts were selected: Chongchuan and Tongzhou (Nantong), and Hailing and Gaogang (Taizhou), considering geographical distribution and urban–rural differences. (2) Streets or towns were randomly selected within each district (four streets in Chongchuan; two streets and two towns in Tongzhou). (3) One community (street) or administrative village (town) was randomly sampled from each selected unit. (4) Eligible family caregivers of disabled older adults were identified through local institutions (community health centers and home care service companies). At least 25 disabled older adults were surveyed per community or village via simple random sampling. Community health centers maintain health records with functional assessments, while home care companies keep registries of disabled older adults receiving services. Researchers obtained lists of eligible caregiver-recipient dyads with staff assistance. Caregiver eligibility was verified by cross-checking disability status against institutional records and confirming primary caregiver status through staff knowledge. Reliance on institutional registration lists, while practical, may have excluded unregistered caregivers not enrolled in formal services, especially those in remote rural areas or families not using public care services. These hidden caregivers may differ systematically from registered ones in socioeconomic status, caregiving intensity, and mental health outcomes, potentially introducing selection bias.

Eligibility criteria for disabled older adults were: (1) receiving in-home care from a family member, and (2) limited independence in daily activities based on the Barthel Index (BI). Exclusion criteria were: (1) residing in nursing homes, and (2) receiving paid in-home care. Caregiver inclusion criteria were: (1) self-identifying as the primary caregiver, (2) providing substantial assistance, and (3) willingness to participate. Caregivers with diagnosed mental disorders or poor compliance were excluded. A priori power analysis using G*Power 3.1 indicated a minimum sample of 135 participants for a small-to-medium effect (*f*^2^ = 0.10–0.15, *α* = 0.05, power = 0.80). Trained interviewers from Nantong University conducted face-to-face structured interviews at caregivers’ homes. Most participants completed the questionnaire independently; for those with limited literacy, interviewers read items aloud in a neutral tone and recorded verbal responses. Each interview lasted 30–45 min. After data collection, researchers reviewed all questionnaires for completeness. Responses with identical scores across all items were excluded. After excluding 13 such questionnaires and 21 with incomplete data, 353 valid questionnaires remained (effective response rate: 91.21%). We adopted a conservative approach by excluding only identical-response patterns across all items, rather than similar scores within subscales, to minimize the risk of excluding genuine responses.

### 2.2 Measurements

#### 2.2.1 Dependent variable

Depressive symptoms were assessed using the 10-item short-form CES-D (Carleton et al., 2013), which is widely validated among middle-aged and older adults in China (Park and Lee, 2021). The scale measures depressive experiences over the past seven days on a four-point Likert scale. Two positive-affect items were reverse-scored. Item scores were summed, with higher scores indicating greater psychological distress. Total scores were analyzed continuously. The scale showed high internal consistency (Cronbach’s *α* = 0.955), with factor loadings from 0.807 to 0.844, CR = 0.955, and AVE = 0.682 (Supplementary Table 3).

#### 2.2.2 Independent variable

Psychological resilience was measured using the 10-item CD-RISC-10 (Connor and Davidson, 2003), which has been well-validated in Chinese populations (Ye et al., 2017). Items are rated on a 5-point Likert scale (0–4), with higher total scores indicating greater resilience. In this study, the scale demonstrated excellent internal consistency (Cronbach’s *α* = 0.953), with factor loadings from 0.804 to 0.840, CR = 0.953, and AVE = 0.670.

#### 2.2.3 Mediating variable

The Zarit Burden Interview (ZBI-12) was employed to measure subjective caregiver burden (Bédard et al., 2001). This instrument comprises 12 items, each scored on a 5-point Likert scale from 0 to 4, where 0 corresponds to “never” and 4 to “almost always”. A total burden score is obtained by summing all item responses, with higher values indicating more severe caregiver burden. The Chinese adaptation of the ZBI-12 followed a standardized forward and back-translation process and has been validated among Chinese caregiver populations, demonstrating satisfactory reliability and validity (Tang et al., 2016; Yu et al., 2018). In the present study, the scale showed strong internal consistency, with a Cronbach’s alpha of 0.943. CFA results showed standardized factor loadings ranging from 0.724 to 0.802, CR of 0.943, and AVE of 0.582.

#### 2.2.4 Moderating variable

The Family APGAR index was originally introduced by Smilkstein and subsequently adapted and translated into Chinese (Qi et al., 2012). Later studies have confirmed that the scale possesses strong reliability and validity within Chinese samples (Huang et al., 2023). The five items cover adaptation, partnership, growth, affection, and resolve, each rated on a 3-point scale (0–2). Total scores range from 0 to 10, with higher scores indicating better family functioning. In this study, the scale showed good reliability (Cronbach’s *α* = 0.895), with factor loadings from 0.771 to 0.822, CR = 0.895, and AVE = 0.631.

#### 2.2.5 Covariates

Covariates included caregivers’ age, sex, marital status, education level, place of residence, employment status, relationship with patients, whether another caregiver, and patient’s years of disability. These variables, which may significantly influence caregivers’ depressive symptoms, were included as covariates in the analytical models (Wang et al., 2026).

### 2.3 Statistical analyses

Statistical analyses were performed using IBM SPSS statistics. Descriptive statistics (means, standard deviations, frequencies, percentages) were computed for participant demographics. Pearson correlation and OLS regression examined relationships among psychological resilience, family functioning, caregiver burden, and depressive symptoms. For exploratory subgroup analyses, participants were stratified by gender, age, marital status, educational attainment, and residence. Linear regression models were estimated with resilience, burden, and family functioning as predictors and depressive symptoms as the outcome. These analyses were not pre-specified and were purely descriptive (see Supplementary Table 2). Results should be interpreted with caution. All control variables were adjusted for across models. Mediation was tested using the bootstrap procedure, with significance determined by whether the 95% CI excluded zero. The moderated mediation model was tested using Hayes’ PROCESS macro (Model 8) for SPSS (Hayes and Preacher, 2014). Model 8 was selected because it allows the moderator to affect both the first stage of the indirect path and the direct path, consistent with our conceptual model (Figure 1) and Hill’s ABC-X model, which posits that family functioning (as resource B) moderates the appraisal of caregiving demands and the prevention of crisis outcomes. Bias-corrected 95% CIs were generated from 5,000 resamples. Moderating effects were further assessed via interaction terms and simple slope tests.

Variance inflation factor values for all predictors ranged from 1.025 to 1.110, all falling well below the conventional threshold of 5, suggesting that multicollinearity was not a concern. Given that self-report data may introduce common method bias (CMB), several precautions were taken. The data collection procedure was carefully designed, and interviewers were thoroughly trained to ensure a solid grasp of the research questions and the survey protocol, thereby improving data accuracy. To statistically assess CMB, Harman’s single-factor test was conducted. The results indicated five factors with eigenvalues exceeding 1. The first factor accounted for 33.68% of the total variance, which is below the recommended cutoff of 40% (Podsakoff et al., 2003). However, it is important to acknowledge that Harman’s single-factor test has been criticized as a relatively insensitive method for detecting common method variance (Jordan and Troth, 2019). The results merely suggest that severe common method variance is unlikely. The procedural precautions taken during data collection further mitigate this concern.

## 3 Results

### 3.1 Sample characteristics

Caregivers in this study had an average age of 64.50 years, with a standard deviation of 11.78. Approximately 63.46% of caregivers were over 60 years of age. Around 62.04% of caregivers were female, and most were married. Only 21.53% of caregivers had attained a high school diploma or higher. Around 18.98% of caregivers were employed, and 33.14% lived in rural areas. Approximately 41.93% of caregivers were spouses of the patients. Most caregivers received support from other family members. Approximately 57.22% of patients had been disabled for five years or less (see Table 1).

**Table 1:** Demographic characteristics of family caregivers and people with disabilities (*n* = 353).

Variables	Mean ± SD	*n* (%)
Age of caregiver	64.50 ± 11.78	
≦60		129 (36.54)
61–70		105 (29.75)
>70		119 (33.71)
Gender of caregiver		
Male		139 (37.96)
Female		219 (62.04)
Marital status		
Married		333 (94.33)
Others		20 (5.67)
Education		
Illiteracy		67 (18.98)
Primary school		99 (28.05)
Middle school		111 (31.44)
High school and above		76 (21.53)
Residence		
Urban		236 (66.86)
Rural		117 (33.14)
Employment status		
Yes		67 (18.98)
No		286 (81.02)
Relation to disabled older adults		
Spouse		148 (41.93)
Children		135 (38.24)
Others		70 (19.83)
Another caregiver		
Yes		291 (82.44)
No		62 (17.56)
Patient’s years of disability		
≦5		202 (57.22)
6–10		89 (25.21)
>10		62 (17.56)

### 3.2 Correlational findings

According to the correlation analysis, psychological resilience was inversely correlated with caregiver burden (*r* = −0.126, *p* < 0.05) and depressive symptoms (*r* = −0.354, *p* < 0.001). Caregiver burden and depressive symptoms were positively associated (*r* = 0.424, *p* < 0.001). Family functioning correlated positively with psychological resilience (*r* = 0.127, *p* < 0.05). Additionally, family functioning was inversely correlated with caregiver burden (*r* = −0.302, *p* < 0.001) and depressive symptoms (*r* = −0.446, *p* < 0.001) (see Table 2).

**Table 2:** Correlations among the key variables.

Variables	Mean ± SD	1	2	3	4
Psychological resilience	23.068 ± 11.132	1			
Caregiver burden	15.844 ± 11.872	−0.126*	1		
Depressive symptoms	10.385 ± 8.528	−0.354***	0.424***	1	
Family functioning	5.867 ± 2.725	0.127*	−0.302***	−0.446***	1

***p < 0.001, **p < 0.01, *p < 0.05.

### 3.3 Mediation analyses

**Figure 2:**
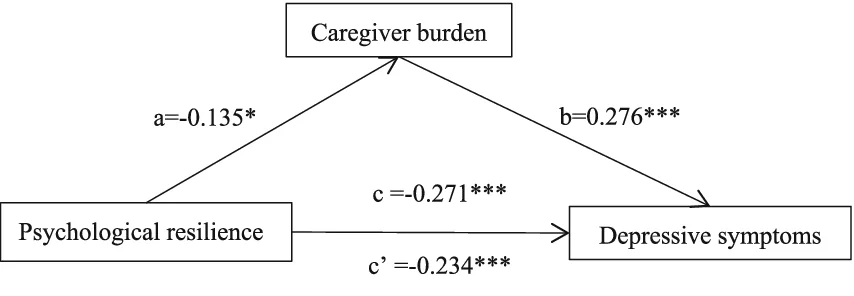
The mediating effect of caregiver burden on the relationship between psychological resilience and depressive symptoms. All covariates were controlled. ***p < 0.001, **p < 0.01, *p < 0.05.

After adjusting for covariates, the outcomes of the mediation model examining caregiver burden are presented in Figure 2 and Supplementary Table 1. The results indicate that psychological resilience was significantly and negatively associated with depressive symptoms (*β* = −0.271, *p* < 0.001). Likewise, psychological resilience was also significantly and negatively associated with caregiver burden (*β* = −0.135, *p* < 0.05). When psychological resilience and caregiver burden are simultaneously entered as predictors of depressive symptoms, psychological resilience remained significantly associated with (*β* = −0.234, *p* < 0.001), whereas caregiver burden was significantly and positively associated with depressive symptoms (*β* = 0.276, *p* < 0.001).

Table 3 indicates that caregiver burden played a partial mediating role in the link between psychological resilience and depressive symptoms. The indirect effect through caregiver burden was estimated at −0.037. The bootstrap confidence interval did not include zero, suggesting a statistically significant indirect effect.

**Table 3:** Bootstrap test of the mediating effects of caregiver burden.

Effect	*β*	SE	LLCI	ULCI
Total effect	−0.271	0.044	−0.357	−0.185
Direct effect	−0.234	0.041	−0.313	−0.154
Indirect effect	−0.037	0.017	−0.070	−0.005

All covariates were controlled. LLCI represents a lower level of the confidence interval, and ULCI represents the upper level of the confidence interval.

### 3.4 Results of moderated mediation analysis

**Figure 3:**
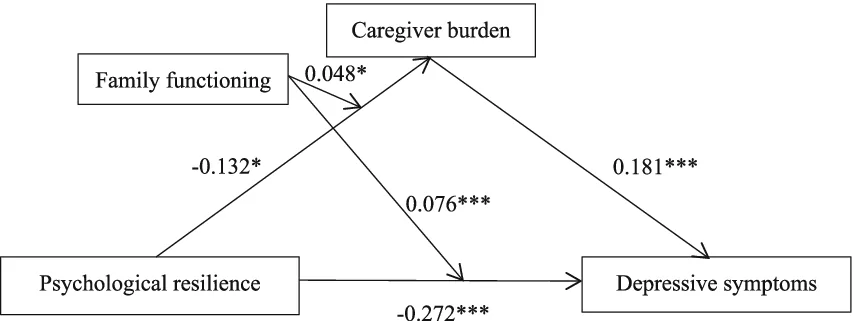
A moderated mediation model of the association between psychological resilience and depressive symptoms through caregiver burden and family functioning. *p < 0.05, ** *p* < 0.01, ***p < 0.001.

Table 4 and Figure 3 show that in Model 1, psychological resilience was significantly associated with caregiver burden (*β* = −0.132, SE = 0.056), with family functioning showing a moderating effect (*β* = 0.048, SE = 0.019). As indicated by Model 2, psychological resilience was significantly associated with depressive symptoms (*β* = −0.272, SE = 0.032), while family functioning played a moderating role (*β* = 0.076, SE = 0.011).

**Table 4:** Moderated mediation regressions of psychological resilience, family functioning, caregiver burden, and depressive symptoms.

Variables	Model 1 (Caregiver burden)			Model 2 (Depressive symptoms)		
	*β*	SE	*t*	*β*	SE	*t*
Psychological resilience	−0.132*	0.056	−2.35	−0.272***	0.032	−8.42
Caregiver burden				0.181***	0.031	5.93
Family functioning	−1.209***	0.222	−5.44	−0.955***	0.132	−7.21
Psychological resilience × Family functioning	0.048*	0.019	2.49	0.076***	0.011	6.87
*R* ^2^	0.115			0.440		
Adj-*R*^2^	0.107			0.434		

All covariates were controlled. *p < 0.05, **p < 0.01, ***p < 0.001.

**Figure 4:**
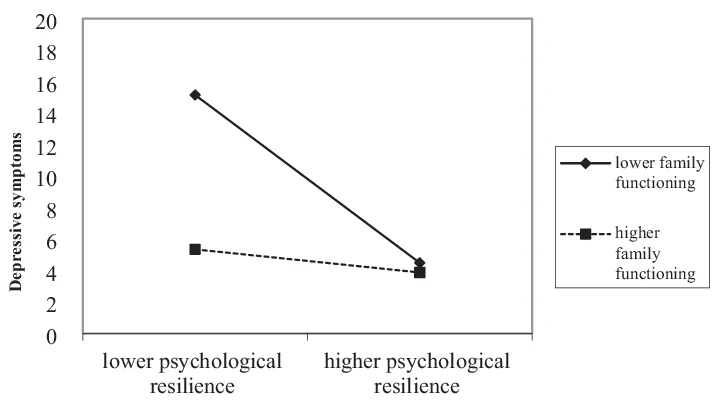
The moderating effect of family functioning on the relationship between psychological resilience and depressive symptoms.

**Figure 5:**
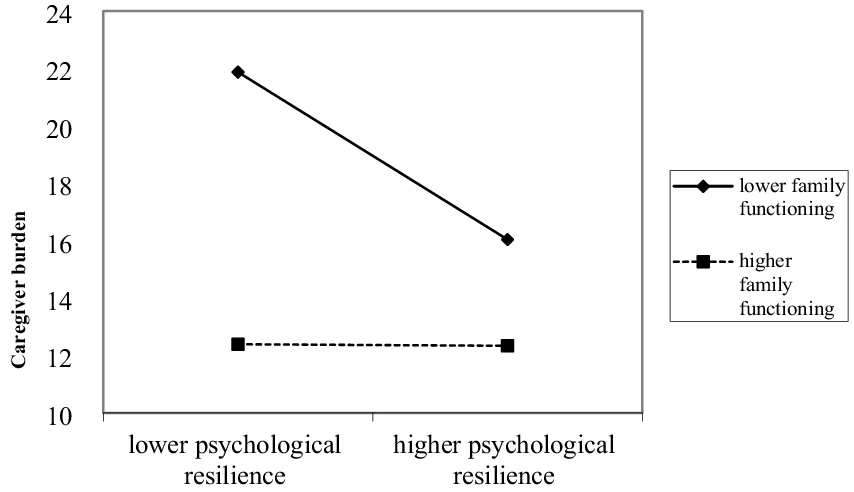
The moderating effect of family functioning on the relationship between psychological resilience and caregiver burden.

The significant interaction was further examined using a simple slope test, with slopes estimated at one standard deviation below and above the family functioning mean. In caregivers reporting lower levels of family functioning, psychological resilience was significantly and negatively associated with depressive symptoms. With higher levels of family functioning, the association between psychological resilience and depressive symptoms was weaker (see Figure 4). Similarly, psychological resilience was significantly associated with caregiver burden only under low family functioning conditions. When family functioning was high, the associations of resilience with both depressive symptoms and caregiver burden were not significant (see Figure 5). To further test the moderating effects, the simple slope estimates were presented in Table 5. The interaction between psychological resilience and family functioning significantly moderated the caregiver burden path at low (−1 SD) and mean levels, but not at the high (+1 SD) level. The index of moderated mediation was significant (95%CI: 0.0018, 0.0174), suggesting that the indirect effect varied across levels of family functioning (see Supplementary Table 4).

**Table 5:** Test for moderating effects.

Interaction items	Moderating effects		95% CI
(Psychological resilience × Family functioning) → Caregiver burden	−1 SD	−0.262	−0.431, −0.093
	M	−0.132	−0.243, −0.022
	+1 SD	−0.003	−0.132, 0.127
(Psychological resilience × Family functioning) → Depressive symptoms	−1 SD	−0.479	−0.577, −0.381
	M	−0.272	−0.336, −0.209
	+1 SD	−0.066	−0.140, 0.008

### 3.5 Additional analysis

Supplementary Table 2 displays the results from the additional subgroup regression analyses. The gender-stratified analysis indicated an inverse association between psychological resilience and depressive symptoms among male (*β* = −0.13, *p* < 0.05) as well as female caregivers (*β* = −0.29, *p* < 0.001). Higher caregiver burden was significantly associated with higher depressive symptoms among caregivers aged ≤60 (*β* = 0.33, *p* < 0.001) and 61–70 (*β* = 0.21, *p* < 0.001), but this relationship was not significant for >70 years [*β* = 0.11, 95%CI (−0.01, 0.23)]. Higher family functioning was significantly associated with lower depressive symptoms in married caregivers [*β* = −1.05, 95%CI (−1.33, −0.76)]. However, for caregivers whose marital status fell into other categories, this association was not statistically significant [*β* = 0.03, 95%CI (−1.52, 1.57)]. When stratified by educational attainment, the link between psychological resilience and depressive symptoms reached non-significance only in the subgroup of caregivers with at least a high school education [*β* = −0.14, 95% CI (−0.28, 0.001)].

## 4 Discussion

A moderated mediation model was put forward in this study to examine how psychological resilience is associated with depressive symptoms through underlying processes. This study identified three key findings: first, a higher degree of psychological resilience was significantly associated with fewer depressive symptoms. Second, caregiver burden served as a partial mediator in the link between psychological resilience and depressive symptoms, suggesting that caregivers with lower resilience reported higher caregiver burden, which in turn was associated with higher depressive symptoms. Third, family functioning moderated this relationship. This moderating effect highlights family functioning’s pivotal role in the stress cascades among caregivers. These findings may contribute to clarifying the risk factors and mechanisms related to depressive symptoms, while also offering insights into how psychological resilience and family functioning may be related to caregiver burden and depressive symptoms within the limitations of a cross-sectional design.

### 4.1 Psychological resilience and depressive symptoms

A notable finding from this study is that caregivers who report higher levels of psychological resilience tend to have substantially fewer depressive symptoms. As a psychological protective factor, resilience has been theorized to enables individuals to recover quickly from negative emotional states (Bajaj and Pande, 2016). This result aligns with existing literature on resilience and depression, in which psychological resilience has been consistently identified as being inversely associated with depressive symptoms (Yan et al., 2025). Resilient individuals are better at using positive coping strategies and regulating emotions under stress, thereby mitigating distress and maintaining emotional balance. In contrast, less resilient individuals are more prone to negative cognitive-emotional patterns, increasing depression risk. As an internal protective resource, resilience reduces the adverse effects of difficulties and promotes adaptation and growth. Moreover, it has been suggested that in China (Yan, 2023), where filial piety norms emphasize familial caregiving obligations, caregivers may perceive their role as inescapable, potentially compounding stress. Caregivers with higher psychological resilience, however, may reframe challenges as manageable or meaningful, which may be associated with lower depression. The practical implications of this finding are significant: it highlights the need to proactively identify caregivers with low psychological resilience at elevated depression risk and strongly supports developing and implementing interventions specifically designed to enhance their psychological resilience in future longitudinal or experimental research.

### 4.2 The mediating role of caregiver burden

Our study additionally identified the mediating role of caregiver burden in the association between psychological resilience and depressive symptoms. This finding contributes to a better understanding of the mechanisms through which psychological resilience is associated with depressive symptoms. Consistent with previous research, higher psychological resilience has been linked to fewer maladaptive psychological states (Chen et al., 2026). Additionally, the present study found that caregiver burden was significantly and positively associated with depressive symptoms, a result consistent with earlier work demonstrating that greater burden is associated with a heightened likelihood of depression (Cao et al., 2026). This finding is consistent with the core tenet of the stress process model (Fergus and Zimmerman, 2005), which posits that chronic stressors, such as long-term care, trigger a series of secondary stressors ultimately associated with adverse mental health outcomes, such as depressive symptoms. Chronic primary stressors, such as long-term care demands, foster secondary stressors like pervasive caregiver burden; without sufficient resilience resources, this burden ultimately may be associated with adverse mental health outcomes.

These findings underscore the need for further research on psychological resilience among caregivers of disabled older adults and for targeted interventions to strengthen it. The results support a partial mediation model, in which caregiver burden accounted for 13.8% of the total association between resilience and depressive symptoms. As the majority of the association remained direct, burden represents one contributing pathway among several, rather than a dominant mechanism. Caregiver depression is likely influenced by multiple factors at personal, interpersonal, and societal levels, including financial strain, care-recipient behaviors, cultural norms, and service access. Therefore, while burden-targeted interventions may offer incremental benefits, especially for subgroups with high burden and low resilience, they should be embedded within broader, multi-component strategies addressing the diverse factors associated with caregiver mental health.

### 4.3 The moderating effects of family functioning

Hypothesis 3 was supported by evidence that family functioning moderates the interrelationships among psychological resilience, caregiver burden, and depressive symptoms. Previous studies have similarly emphasized the role of family functioning in depression among caregivers of older adults with disabilities, which is consistent with the tenets of ecological systems theory (Bronfenbrenner, 1977), which posits that environmental elements including family and peers are associated with psychological wellbeing. The role of family functioning in the development of depression has been well documented in earlier studies (Shao et al., 2020). Strong family structures have been associated with caregivers’ ability to manage external adverse life events effectively, which in turn has been associated with lower depression risk (Jiayuan et al., 2026). Dysfunctional family environments have been linked to vulnerability to stressors, and depressive symptoms.

A noteworthy finding is that the association of psychological resilience with both caregiver burden and depressive symptoms was more pronounced under low family functioning than under high family functioning. This pattern suggests that resilience serves as a compensatory resource, particularly salient when external family support is limited. According to resource substitution theory (Ross et al., 2001), when external resources such as family cohesion are scarce, individuals rely more heavily on internal resources like resilience to manage caregiving stress. Conversely, when family functioning is high, external support may partially substitute for internal resilience, diminishing its incremental association. In other words, family functioning moderates the resilience-burden relationship: resilience is more strongly associated with lower burden when family resources are limited, and this association weakens as family functioning improves. A similar pattern was observed for the resilience-depression relationship. Family functioning moderated this pathway such that resilience was more protective against depressive symptoms under low family functioning. When caregivers face poor family relationships or weak cohesion, resilience serves as a critical internal resource. However, when family functioning is strong, caregivers receive sufficient emotional support from family members, reducing the need for resilience to protect against depression. This buffering pattern aligns with the stress-buffering hypothesis (Cohen and Wills, 1985), which suggests that supportive social relationships mitigate the adverse effects of stress. Thus, family functioning not only directly associates with lower depression, but also alters the conditions under which resilience is protective.

This compensatory mechanism is also consistent with family systems theory, which posits that individuals operate within nested ecological systems. When the immediate family microsystem provides insufficient support, internal resources become more critical for navigating stressors. Our findings extend this perspective by suggesting that the interplay between internal and external resources is interactive rather than merely additive. Resilience is not simply associated with family support; its association with outcomes depends on the level of family functioning. This pattern also aligns with Hill’s ABC-X model, in which caregiving demands represent the stressor (A), resources including both internal resilience and external family functioning represent the resources (B), and depressive symptoms are viewed as the crisis outcome (X). When family functioning is poor, individual psychological resources become more critical in determining whether caregiving stress escalates to a crisis. The significant interaction across both pathways suggests that resources at different levels of the ecological system interact to influence mental health outcomes, rather than operating independently.

These findings have practical implications. For caregivers in families with limited functioning, strengthening resilience may be particularly beneficial, as internal resources become the primary buffer against burden and depressive symptoms. For those in well-functioning families, resilience-building efforts may yield smaller additional benefits, as the family context already provides substantial protection. This suggests that interventions should be tailored according to family functioning level. This study identifies caregiver burden as a key factor associated with depressive symptoms, suggesting that efforts to address burden warrant high priority. Furthermore, the results highlight the importance of intervention programs aimed at strengthening family functioning. Healthcare providers and community workers should focus on family dynamics and consider targeted interventions to address depressive symptoms in this population.

### 4.4 Exploratory subgroup analyses

We conducted exploratory subgroup analyses stratified by gender, age, marital status, and educational attainment. The resilience-depression association appeared stronger in females. This may reflect gender differences in depression-anxiety symptom networks, where females exhibit distinct symptom connectivity patterns (Yang et al., 2026). Caregiver burden was associated with depressive symptoms only among caregivers aged ≤70, consistent with socioemotional selectivity theory, which suggests that older adults develop enhanced emotion regulation (Carstensen, 2021). Family functioning was linked to lower depressive symptoms only in married caregivers, as spousal support may offer more consistent protection (Huang et al., 2023). The resilience-depression association was non-significant among caregivers with higher education, suggesting education may provide alternative coping resources (Yan et al., 2025).

### 4.5 Strengths and limitations

This study represents an investigation into the underlying mechanisms through which psychological resilience is associated with depressive symptoms via caregiver burden, while simultaneously examining the dual moderating effects of family functioning. While previous research has mainly examined simple mediation or isolated moderation effects, the combined moderated mediation approach provides a deeper understanding of the interplay between family systems and personal resilience. Furthermore, the findings offer actionable insights for developing interventions.

Nevertheless, several limitations warrant acknowledgment. First, the cross-sectional design precludes causal inferences. Longitudinal studies are needed to establish temporal dynamics. Second, the model explained a relatively modest proportion of variance in depressive symptoms, suggesting that other unmeasured factors contribute to caregiver mental health. Future research should integrate additional variables to enhance explanatory power. Third, self-reported data may be subject to recall bias. Multi-method approaches should be considered in future work. Fourth, the sample was restricted to Nantong and Taizhou in Jiangsu Province, limiting generalizability. Nantong is a recognized longevity region with higher life expectancy, which may entail longer caregiving durations. Jiangsu has notable intra-provincial disparities: southern Jiangsu is highly developed with well-established elderly welfare systems, whereas central and northern Jiangsu lag behind in GDP and public care resources. Even larger gaps exist across central, western, and northern provinces. Thus, findings should be generalized cautiously, and future research should include diverse provincial samples. Fifth, reliance on institutional registration lists may have excluded unregistered caregivers not enrolled in formal services. These hidden caregivers may differ systematically from registered ones in socioeconomic status, caregiving intensity, and mental health outcomes. Although we adjusted for observed covariates, unmeasured differences remain a concern. Future studies should employ supplementary recruitment strategies, such as snowball sampling or household surveys, to reach this population. Finally, the demographic profile of our sample (e.g., older age, lower education, predominance of spousal caregivers) reflects the reality of the specific community setting but also necessitates caution when extending the conclusions to other caregiver subgroups, such as younger generations, highly educated individuals, or non-spousal caregivers. Future research is required to replicate these findings across more diverse caregiver populations to build a more comprehensive understanding. In addition, future research should extend these findings by examining two underexplored areas. First, the compounding effects of multiple chronic conditions among disabled older adults on caregiver depression warrant further investigation. Second, gender heterogeneity in depressive symptom mechanisms deserves greater empirical attention. Network analysis studies have revealed that the structure and connectivity of depression-anxiety symptoms differ significantly between males and females (Yang et al., 2026; You et al., 2026). Longitudinal and network-based approaches could further clarify how gender moderates the pathways identified in the present study.

## 5 Conclusion

This study provides preliminary findings regarding how caregiver burden mediates, and family functioning moderates, the relationship between psychological resilience and depressive symptoms. Future investigations may consider intervention strategies that target caregiver burden reduction and family functioning enhancement to address caregivers’ depressive symptoms. Furthermore, given the observed association between family functioning and depressive symptoms, healthcare providers could emphasize the importance of social and family support resources to assist in addressing the psychological difficulties experienced by caregivers.

## Data Availability

The raw data supporting the conclusions of this article will be made available by the authors, without undue reservation.

## References

[ref1] Andrade J. J. D. C. Silva A. C. O. Fraz O I. D. S. Perrelli J. G. A. Silva T. T. D. M. Cavalcanti A. M. T. S. (2021). Family functionality and burden of family caregivers of users with mental disorders. Rev. Bras. Enferm. 74:e20201061. doi: 10.1590/0034-7167-2020-1061 34468548

[ref2] Awano N. Oyama N. Akiyama K. Inomata M. Kuse N. Tone M. . (2020). Anxiety, depression, and resilience of healthcare workers in Japan during the coronavirus disease 2019 outbreak. Intern. Med. (Tokyo, Japan) 59, 2693–2699. doi: 10.2169/internalmedicine.5694-20, 33132305 PMC7691033

[ref3] Bajaj B. Pande N. (2016). Mediating role of resilience in the impact of mindfulness on life satisfaction and affect as indices of subjective wellbeing. Pers. Individ. Differ. 93, 63–67. doi: 10.1016/j.paid.2015.09.005

[ref4] Bédard M. Molloy D. W. Squire L. Dubois S. Lever J. A. O'Donnell M. (2001). The Zarit burden interview: a new short version and screening version. Gerontologist 41, 652–657. doi: 10.1093/geront/41.5.652, 11574710

[ref5] Bogner H. R. Morales K. H. Reynolds C. F. Cary M. S. Bruce M. L. (2012). Prognostic factors, course, and outcome of depression among older primary care patients: the PROSPECT study. Aging Ment. Health 16, 452–461. doi: 10.1080/13607863.2011.638904, 22296508 PMC3323766

[ref6] Brik A. B. Wang Y. (2025). Exploring the factors contributing to parent stress symptoms during the COVID-19 pandemic in Europe: an ABC-X model approach. Fam. Process 64:e13063. doi: 10.1111/famp.13063, 39334528 PMC11786249

[ref7] Bronfenbrenner U. (1977). Toward an experimental ecology of human development. Am. Psychol. 32, 513–531. doi: 10.1037/0003-066X.32.7.513

[ref8] Cao X. (2019). Research on multi-subject fusion mechanism of long-term care financial supply in China. Chin. Public Adm., 107–112. doi: 10.19735/j.issn.1006-0863.2019.09.16

[ref9] Cao K. Q. Madden E. B. Graven L. J. (2026). Caregiving burden and depressive symptoms in aphasia caregivers: the moderating role of friendship support. Am. J. Speech Lang. Pathol. 35, 787–799. doi: 10.1044/2025_AJSLP-25-00367 41734209

[ref10] Carleton R. N. Thibodeau M. A. Teale M. J. N. Welch P. G. Abrams M. P. Robinson T. . (2013). The center for epidemiologic studies depression scale: a review with a theoretical and empirical examination of item content and factor structure. PLoS One 8:e58067. doi: 10.1371/journal.pone.0058067, 23469262 PMC3585724

[ref11] Carstensen L. L. (2021). Socioemotional selectivity theory: the role of perceived endings in human motivation. Gerontologist 61, 1188–1196. doi: 10.1093/geront/gnab116, 34718558 PMC8599276

[ref12] Charbaji S. Ei Asmar K. McCall S. J. Saad G. E. Chaaya M. (2026). Caregiver burden and mental health among informal caregivers of older adults in Lebanon. Discov. Public Health 23:114. doi: 10.1186/s12982-026-01466-4, 41630982 PMC12860812

[ref13] Chen S. Li S. Zhang Z. (2026). The relationship between perceived stress, psychological resilience, and depressive symptoms in college students, and the moderating role of gender. Sci. Rep. 16:12789. doi: 10.1038/s41598-026-43237-w, 41803231 PMC13096648

[ref14] Cohen S. Wills T. A. (1985). Stress, social support, and the buffering hypothesis. Psychol. Bull. 98, 310–357. doi: 10.1037/0033-2909.98.2.310, 3901065

[ref15] Connor K. M. Davidson J. R. T. (2003). Development of a new resilience scale: the Connor-Davidson Resilience Scale (CD-RISC). Depress. Anxiety 18, 76–82. doi: 10.1002/da.10113, 12964174

[ref16] Cui P. Yang M. Hu H. Cheng C. Chen X. Shi J. . (2024). The impact of caregiver burden on quality of life in family caregivers of patients with advanced cancer: a moderated mediation analysis of the role of psychological distress and family resilience. BMC Public Health 24:817. doi: 10.1186/s12889-024-18321-3, 38491454 PMC10941369

[ref17] Dang Y. Zhang L. Meng Z. Qi H. Yang M. (2026). The cyclical relationship between family functioning and depressive symptoms in Chinese children: longitudinal mediating effects of parent-child attachment. Curr. Psychol. 45:997. doi: 10.1007/s12144-026-09571-y

[ref18] Del-Pino-Casado R. Rodríguez Cardosa M. López-Martínez C. Orgeta V. (2019). The association between subjective caregiver burden and depressive symptoms in carers of older relatives: a systematic review and meta-analysis. PLoS One 14. doi: 10.1371/journal.pone.0217648, 31141556 PMC6541277

[ref19] Desjardins L. Solomon A. Shama W. Mills D. Chung J. Hancock K. . (2022). The impact of caregiver anxiety/depression symptoms and family functioning on child quality of life during pediatric cancer treatment: from diagnosis to 6 months. J. Psychosoc. Oncol. 40, 790–807. doi: 10.1080/07347332.2021.2015646, 35016592

[ref20] Fergus S. Zimmerman M. A. (2005). Adolescent resilience: a framework for understanding healthy development in the face of risk. Annu. Rev. Public Health 26, 399–419. doi: 10.1146/annurev.publhealth.26.021304.144357, 15760295

[ref21] Guerrero-Mu Oz D. Salazar D. Constain V. Perez A. Pineda-Ca Ar C. A. García-Perdomo H. A. (2021). Association between family functionality and depression: a systematic review and meta-analysis. Korean J. Fam. Med. 42, 172–180. doi: 10.4082/kjfm.19.0166, 32521579 PMC8010447

[ref22] Hagedoorn M. T. Sanderman R. Bolks H. N. Tuinstra J. Coyne J. C. (2008). Distress in couples coping with cancer: a meta-analysis and critical review of role and gender effects. Psychol. Bull. 134, 1–30. doi: 10.1037/0033-2909.134.1.1, 18193993

[ref23] Hayes A. F. Preacher K. J. (2014). Statistical mediation analysis with a multicategorical independent variable. Br. J. Math. Stat. Psychol. 67, 451–470. doi: 10.1111/bmsp.12028, 24188158

[ref24] Hill R. (1958). Social stresses on the family. Soc. Casework 39, 139–150. doi: 10.1177/1044389458039002-318

[ref25] Huang A. Liu L. Wang X. Chen J. Liang S. Peng X. . (2023). Intolerance of uncertainty and anxiety among college students during the re-emergence of COVID-19: mediation effects of cognitive emotion regulation and moderation effects of family function. J. Affect. Disord. 327, 378–384. doi: 10.1016/j.jad.2023.01.110, 36764364 PMC9908432

[ref26] Ji Q. Zhang L. Ji P. Song M. Xu J. Chen Y. . (2025). The relationship between psychological resilience and quality of life among primary caregivers of cancer patients: the mediating role of care burden and the moderating role of social support. Support. Care Cancer. 33:343. doi: 10.1007/s00520-025-09412-x, 40172687

[ref27] Jiao N. Luo S. Xie Y. Zhang B. Wang S. Shen Y. (2023). Analysis of mental health status and influencing factors of caregivers of disabled elderly at home. Mod. Prev. Med. 50, 4182–4187. doi: 10.20043/j.cnki.MPM.202303215

[ref28] Jiayuan Z. Hui Z. Yang L. Yuqiu Z. (2026). The effect of family functioning on depressive symptoms in adolescents: a moderated mediation model. Depress. Anxiety 2026:9965173. doi: 10.1155/da/9965173, 41635674 PMC12861822

[ref29] Jordan P. J. Troth A. C. (2019). Common method bias in applied settings: the dilemma of researching in organizations. Aust. J. Manag. 45, 3–14. doi: 10.1177/0312896219871976

[ref30] Kara Ar B. Canli D. (2020). Psychological resilience and depression during the Covid-19 pandemic in Turkey. Psychiatr. Danub. 32, 273–279. doi: 10.24869/psyd.2020.273 32796798

[ref31] Kim Y. Carver C. S. (2012). Recognizing the value and needs of the caregiver in oncology. Curr. Opin. Support. Palliat. Car. 6, 280–288. doi: 10.1097/SPC.0b013e3283526999, 22436321

[ref32] Kumpfer K. L. (2002). “Factors and processes contributing to resilience,” in Resilience and Development: Positive Life Adaptations. eds. Glantz M. D. Johnson J. L. (Boston: Springer), 179–224. doi: 10.1007/0-306-47167-1_9

[ref33] Li R. Cooper C. Livingston G. (2014). Relationship of coping style to mood and anxiety disorders in dementia carers. Curr. Opin. Psychiatry 27, 52–56. doi: 10.1097/YCO.0000000000000020, 24270483

[ref34] Li J. Kong X. Wang J. Zhu H. Zhong J. Cao Y. . (2023). Family functioning and patients' depressive symptoms: comparison in perceived family function between patients who had an acute ischaemic stroke and their primary family caregivers—a cross-sectional study. BMJ Open 13:e68794. doi: 10.1136/bmjopen-2022-068794, 37989357 PMC10668298

[ref35] Li S. Xu Q. Xie J. Wang L. Li H. Ma L. . (2022). Associations of parenting daily hassles with parents' mental health during the COVID-19 school closure. Soc. Sci. Med. 311:115301. doi: 10.1016/j.socscimed.2022.115301 36063592 PMC9422338

[ref36] Lin D. Liao J. Liu Y. Zhong Y. Pan J. Fang H. . (2025). Analysis of factors influencing the burden on family caregivers of disabled older adults in Guangzhou. Front. Public Health 13:1628022. doi: 10.3389/fpubh.2025.1628022, 40917419 PMC12408277

[ref37] Liu X. Li J. Yao J. Zhang L. (2026). Mediating effect of family functioning on depressive symptoms and family resilience in dementia caregivers. Front. Public Health 14:1784746. doi: 10.3389/fpubh.2026.1784746, 42200121 PMC13199328

[ref38] Liu H. Liu X. Liu Z. Wang Y. Feng R. Zheng R. . (2022). Death anxiety and its relationship with family function and meaning in life in patients with advanced cancer—a cross-sectional survey in China. Asia-Pac. J. Oncol. Nurs. 9:100134. doi: 10.1016/j.apjon.2022.100134, 36204085 PMC9529665

[ref39] Margolin G. Gordis E. B. (2000). The effects of family and community violence on children. Annu. Rev. Psychol. 51, 445–479. doi: 10.1146/annurev.psych.51.1.445 10751978

[ref40] Niu Y. Xu Z. Huang J. Guo S. Lou T. Bai X. . (2025). A latent class analysis of resilience and its relationship with care burden and psychological distress in family caregivers of older adults with disability. Nurs. Health Sci. 27:70069. doi: 10.1111/nhs.70069, 40064541

[ref41] Park S. Lee H. (2021). Is the center for epidemiologic studies depression scale as useful as the geriatric depression scale in screening for late-life depression? A systematic review. J. Affect. Disord. 292, 454–463. doi: 10.1016/j.jad.2021.05.120, 34144371

[ref42] Parviniannasab A. M. Faramarzian Z. Hosseini S. A. Hamidizadeh S. Bijani M. (2024). The effect of social support, diabetes management self-efficacy, and diabetes distress on resilience among patients with type 2 diabetes: a moderated mediation analysis. BMC Public Health 24:477. doi: 10.1186/s12889-024-18022-x, 38360647 PMC10868118

[ref43] Pearlin L. I. Mullan J. T. Semple S. J. Skaff M. M. (1990). Caregiving and the stress process: an overview of concepts and their measures. Gerontologist 30, 583–594. doi: 10.1093/geront/30.5.583, 2276631

[ref44] Peng Y. Zhang S. Wang M. Sheng K. Peng Q. Zhang G. (2019). A study on the psychological resilience of primary caregivers of disabled elderly and its correlation with care burden and quality of life. J. Nurs. Train. 34, 909–912.

[ref45] Pinquart M. Sörensen S. (2003). Associations of caregiver stressors and uplifts with subjective wellbeing and depressive mood: a meta-analytic comparison. Aging Ment. Health 8, 438–449. doi: 10.1080/13607860410001725036, 15511742

[ref46] Pires C. Borim F. S. A. Queluz F. Cachioni M. (2022). Burden, family functioning, and psychological health of older caregivers of older adults: a path analysis. Geriatr. Gerontol. Aging 16:e0220022. doi: 10.53886/gga.e0220022

[ref47] Podsakoff P. M. MacKenzie S. B. Lee J. Podsakoff N. P. (2003). Common method biases in behavioral research: a critical review of the literature and recommended remedies. J. Appl. Psychol. 88, 879–903. doi: 10.1037/0021-9010.88.5.879, 14516251

[ref48] Qi H. Jia C. Zhang Z. Feng X. Zhang J. (2012). Correlation between family care level and quality of life in coronary artery disease patients with heart dysfunction. Chin. Gen. Pract. 15, 834–836.

[ref49] Qiu F. X. Zhan H. J. Liu J. Barrett P. M. (2020). Downward transfer of support and care: understanding the cultural lag in rural China. Ageing Soc. 42, 1422–1447. doi: 10.1017/s0144686x2000152x

[ref50] Qu T. Wang S. Wang K. Mao F. Li M. (2022). Qualitative research on the experience and hierarchy of needs of primary caregiver of disabled elderly. Med. Philos. 43, 57–61. doi: 10.12014/j.issn.1002-0772.2022.17.12

[ref51] Ren L. Zhang Y. Yang F. Zhang Y. (2017). Investigation on caregiver burden and depression among main caregivers of long-term coma patients. Clin. Res. Pract. 2, 176–178. doi: 10.19347/j.cnki.2096-1413.201706092

[ref52] Ross C. E. Mirowsky J. Pribesh S. (2001). Powerlessness and the amplification of threat: neighborhood disadvantage, disorder, and mistrust. Am. Sociol. Rev. 66, 568–591. doi: 10.2307/3088923

[ref53] Shao R. He P. Ling B. Tan L. Xu L. Hou Y. . (2020). Prevalence of depression and anxiety and correlations between depression, anxiety, family functioning, social support and coping styles among Chinese medical students. BMC Psychol. 8:38. doi: 10.1186/s40359-020-00402-8, 32321593 PMC7178943

[ref54] Tang J. Y. Ho A. H. Luo H. Wong G. H. Lau B. H. Lum T. Y. . (2016). Validating a Cantonese short version of the Zarit burden interview (CZBI-short) for dementia caregivers. Aging Ment. Health 20, 996–1001. doi: 10.1080/13607863.2015.1047323, 26016419

[ref55] Tang X. Zhao S. Zhang M. Zhou J. Wang Y. He B. (2022). Effects of disability severity on the family burden of home-dwelling Uygur and Kazakh aged in rural Western China: family function as a mediator. J. Transcult. Nurs. 33, 511–520. doi: 10.1177/10436596221090271, 35481759

[ref56] Tugade M. M. Fredrickson B. L. (2004). Psychological resilience and positive emotional granularity: examining the benefits of positive emotions on coping and health. J. Pers. Soc. Psychol. 86, 320–333. doi: 10.1037/0022-3514.86.2.320, 15509280 PMC1201429

[ref57] Wang L. Zhang X. Cheng S. Deng K. (2026). Development and validation of a risk screening model for depressive symptoms in older inpatients: a cross-sectional study. Clin. Interv. Aging 21, 1–17. doi: 10.2147/cia.s599044, 42165005 PMC13185958

[ref58] Wang J. Zhang J. Lin H. Le Chang Tu J. Tang J. . (2024). Come together! Population aggregation, strong tie, and individual depression: a moderated mediating investigation. Curr. Psychol. 43, 34319–34334. doi: 10.1007/s12144-024-06969-4

[ref59] Wrede N. T Pfer N. F. Wilz G. Pfeiffer K. (2025). Decomposing the association between caregiver burden and depressive symptoms in family caregivers of older adults: a network analysis. J. Affect. Disord. 387:119547. doi: 10.1016/j.jad.2025.119547 40441656

[ref60] Wu Y. Zhao X. Ding X. Yang H. Qian Z. Feng F. . (2017). A prospective study of psychological resilience and depression among left-behind children in China. J. Health Psychol. 22, 627–636. doi: 10.1177/1359105315610811, 26490625

[ref61] Yan Y. (2023). Familial affections Vis-à-Vis filial piety: the ethical challenges facing eldercare under neo-familism in contemporary China. J. Chin. Sociol. 10:5. doi: 10.1186/s40711-023-00185-6

[ref62] Yan Y. Duan X. Tan Y. Wu T. Yang B. X. Luo D. . (2025). The relationship between family functioning and depressive symptoms: mediating effects of psychological resilience and parent-child interactions. J. Affect. Disord. 385:119383. doi: 10.1016/j.jad.2025.119383, 40347543

[ref63] Yang M. B. Wu Y. X. Zhang W. X. Liu H. Y. Lin H. Xi M. . (2026). Gender-specific depression-anxiety symptom networks and the impact of weight status: insights from a large-scale study. PsyCh J. 15:e70104. doi: 10.1002/pchj.70104, 42210799 PMC13240150

[ref64] Ye Z. J. Qiu H. Z. Li P. F. Chen P. Liang M. Z. Liu M. L. . (2017). Validation and application of the Chinese version of the 10-item Connor-Davidson Resilience Scale (CD-RISC-10) among parents of children with cancer diagnosis. Eur. J. Oncol. Nurs. 27, 36–44. doi: 10.1016/j.ejon.2017.01.004, 28279394

[ref65] Yeh K. H. Bedford O. (2003). A test of the dual filial piety model. Asian J. Soc. Psychol. 6, 215–228. doi: 10.1046/j.1467-839x.2003.00122.x

[ref66] You S. Lee J. Lee Y. Chun J. (2026). Sex differences in empathy, emotional expressiveness, and conflict resolution among upper elementary school students. Pers. Individ. Differ. 262:113953. doi: 10.1016/j.paid.2026.113953

[ref67] Yu Y. Liu Z. Zhou W. Chen X. Zhang X. Hu M. . (2018). Assessment of burden among family caregivers of schizophrenia: psychometric testing for short-form Zarit burden interviews. Front. Psychol. 9:2539. doi: 10.3389/fpsyg.2018.02539, 30618960 PMC6305706

[ref68] Zhang M. Gao H. (2023). A survey on the status quo and relationship of family function, depression and psychological resilience among adolescent students. J. Hefei Univ. 40, 139–144. doi: 10.3969/j.issn.1673-162X.2023.04.023

[ref69] Zhang J. Sun X. Yan Z. (2024). Blessing or curse: the role of authoritarian filial piety and self-efficacy in caregiver gains among Chinese family caregivers caring for physically impaired older adults. BMC Geriatr. 24:163. doi: 10.1186/s12877-024-04768-x, 38365573 PMC10870663

[ref70] Zhang J. Zhang D. Xue X. Wang X. Ding S. Ma Y. (2025). Intergenerational psychological capital, disability, and depressive symptoms in the shadow of functional deprivation among middle-aged and older adults in China. Psychol. Res. Behav. Manag. 18, 2237–2257. doi: 10.2147/PRBM.S552384, 41181515 PMC12579458

[ref71] Zhao L. (2023). China's aging population: a review of living arrangement, intergenerational support, and wellbeing. Health Care Sci. 2, 317–327. doi: 10.1002/hcs2.64, 38938584 PMC11080716

[ref72] Zhu W. Gu R. Wang Y. (2026). Family functioning and depression in Chinese nursing home residents: the serial mediating role of perceived stress and psychological resilience. Clin. Gerontol. 49, 388–401. doi: 10.1080/07317115.2025.2556032, 40905447

