# Supplementary material for: Psychological resilience and depressive symptoms among family caregivers of disabled people: the mediating role of caregiver burden and the moderating role of family functioning
Source: Front Psychol. 2026 Aug 14;17:1852506. doi: 10.3389/fpsyg.2026.1852506 (PMC13521876; doi:10.3389/fpsyg.2026.1852506)
Supplement: Supplementary file 1 [file Table_1.DOCX]

**Supplementary Table 1** Mediation models of psychological resilience on depressive symptoms through caregiver burden

| Dependent variable | Independent variable | *β* | SE | R^2^ | Adj-R^2^ |
| --- | --- | --- | --- | --- | --- |
| Depressive symptoms | Psychological resilience | -0.271*** | 0.038 | 0.125 | 0.123 |
| Caregiver burden | Psychological resilience | -0.135* | 0.056 | 0.016 | 0.013 |
| Depressive symptoms | Psychological resilience | -0.234*** | 0.035 | 0.271 | 0.267 |
|  | Caregiver burden | 0.276*** | 0.033 |  |  |

Note: All covariates were controlled. **P*<0.05, ** *P*<0.01, *** *P*<0.001

**Supplementary Table 2** The subgroup analysis of basic characteristics

| Variables | | Psychological resilience | Caregiver burden | Family functioning | Adj-R^2^ |
| --- | --- | --- | --- | --- | --- |
| Gender | Male | -0.13(-0.23,-0.03)^*^ | 0.14(0.03,0.25)^*^ | -1.31(-1.76,-0.85)^***^ | 0.29 |
|  | Female | -0.29(-0.37,-0.20)^***^ | 0.24(0.17,0.32)^***^ | -0.74(-1.09,-0.40)^***^ | 0.42 |
| Age | ≦60 | -0.22(-0.31,-0.13)^***^ | 0.33(0.23,0.43)^***^ | -0.60(-1.01,-0.19)^**^ | 0.45 |
|  | 61-70 | -0.15(-0.27,-0.03)^*^ | 0.21(0.10,0.32)^***^ | -1.47(-1.95,-1.00)^***^ | 0.42 |
|  | >70 | -0.25(-0.38,-0.12)^***^ | 0.11(-0.01,0.23) | -0.92(-1.48,-0.36)^**^ | 0.25 |
| Marital status | Unmarried | -0.16(-0.50,-0.18) | 0.35(0.12,0.58)^**^ | 0.03(-1.52,1.57) | 0.40 |
|  | Married | -0.21(-0.28,-0.14)^***^ | 0.20(0.13,0.27)^***^ | -1.05(-1.33,-0.76)^***^ | 0.36 |
| Education | Illiteracy | -0.31(-0.48,-0.15)^***^ | 0.20(0.07,0.34)^**^ | -0.79(-1.43,-0.16)^*^ | 0.43 |
|  | Primary school | -0.18(-0.32,-0.04)^*^ | 0.15(0.02,0.28)^*^ | -1.38(-2.00,-0.75)^***^ | 0.31 |
|  | Middle school | -0.24(-0.34,-0.14)^***^ | 0.30(0.18,0.41)^***^ | -0.69(-1.12,-0.26)^**^ | 0.43 |
|  | High school and above | -0.14(-0.28,0.00) | 0.17(0.03,0.31)^*^ | -1.20(-1.84,-0.56)^***^ | 0.26 |
| residence | Urban | -0.22(-0.30,-0.14)^***^ | 0.19(0.11,0.26)^***^ | -1.08(-1.43,-0.74)^***^ | 0.35 |
|  | Rural | -0.18(-0.30,-0.07)^**^ | 0.27(0.16,0.38)^***^ | -0.82(-1.29,-0.34)^***^ | 0.38 |

Note: ^*^ *P* < 0.05, ^**^*P* < 0.01, ^***^*P* < 0.001.

95%CI = Bootstrap confidence intervals with lower and upper limits.

Caregivers’ age, sex, marital status, education level, place of residence, employment status, relationship with patients, whether another caregiver, and patient’s years of disability were analyzed as control variables.

**Supplementary Table 3** Indicators and loading factors in the measurement models

| Measurement models | Indicators | Unstandardised  factor loadings | SE | Z | Standardised  factor loadings | AVE | CR |
| --- | --- | --- | --- | --- | --- | --- | --- |
| Psychological resilience | f01 | 1.000 |  |  | 0.817 | 0.670 | 0.953 |
|  | f02 | 1.046 | 0.055 | 18.901 | 0.836 |  |  |
|  | f03 | 0.973 | 0.055 | 17.833 | 0.804 |  |  |
|  | f04 | 0.920 | 0.051 | 17.894 | 0.806 |  |  |
|  | f05 | 0.954 | 0.053 | 17.909 | 0.806 |  |  |
|  | f06 | 0.970 | 0.053 | 18.251 | 0.817 |  |  |
|  | f07 | 1.016 | 0.053 | 19.041 | 0.840 |  |  |
|  | f08 | 0.940 | 0.051 | 18.376 | 0.821 |  |  |
|  | f09 | 1.000 | 0.054 | 18.599 | 0.827 |  |  |
|  | f10 | 0.960 | 0.053 | 18.098 | 0.812 |  |  |
| Depressive symptoms | h01 | 1.000 |  |  | 0.807 | 0.682 | 0.955 |
|  | h02 | 1.083 | 0.059 | 18.328 | 0.829 |  |  |
|  | h03 | 1.078 | 0.059 | 18.211 | 0.826 |  |  |
|  | h04 | 1.098 | 0.060 | 18.334 | 0.830 |  |  |
|  | h05 | 1.115 | 0.059 | 18.799 | 0.844 |  |  |
|  | h06 | 1.071 | 0.059 | 18.233 | 0.826 |  |  |
|  | h07 | 1.120 | 0.060 | 18.618 | 0.838 |  |  |
|  | h08 | 1.053 | 0.058 | 18.278 | 0.828 |  |  |
|  | h09 | 1.056 | 0.059 | 18.021 | 0.820 |  |  |
|  | h10 | 1.049 | 0.059 | 17.680 | 0.809 |  |  |
| Caregiver burden | i01 | 1.000 |  |  | 0.782 | 0.582 | 0.943 |
|  | i02 | 0.991 | 0.063 | 15.639 | 0.765 |  |  |
|  | i03 | 0.867 | 0.059 | 14.609 | 0.724 |  |  |
|  | i04 | 0.927 | 0.059 | 15.590 | 0.763 |  |  |
|  | i05 | 0.935 | 0.061 | 15.273 | 0.751 |  |  |
|  | i06 | 0.941 | 0.062 | 15.210 | 0.748 |  |  |
|  | i07 | 1.016 | 0.064 | 15.936 | 0.777 |  |  |
|  | i08 | 0.967 | 0.063 | 15.349 | 0.754 |  |  |
|  | i09 | 0.947 | 0.061 | 15.443 | 0.758 |  |  |
|  | i10 | 1.051 | 0.063 | 16.611 | 0.802 |  |  |
|  | i11 | 1.002 | 0.064 | 15.740 | 0.769 |  |  |
|  | i12 | 0.915 | 0.059 | 15.401 | 0.756 |  |  |
| Family functioning | k01 | 1.000 |  |  | 0.822 | 0.631 | 0.895 |
|  | k02 | 0.895 | 0.056 | 16.122 | 0.779 |  |  |
|  | k03 | 1.001 | 0.059 | 16.912 | 0.808 |  |  |
|  | k04 | 0.949 | 0.058 | 16.435 | 0.790 |  |  |
|  | k05 | 0.916 | 0.058 | 15.907 | 0.771 |  |  |

**Supplementary Table 4** Index of moderated mediation

| Variable | Index | 95%CI |
| --- | --- | --- |
| Family functioning | 0.009 | 0.002, 0.017 |
